# Creating value through outreach in a hospital setting: a case study from Zuckerberg San Francisco General Hospital Library

**DOI:** 10.5195/jmla.2018.454

**Published:** 2018-10-01

**Authors:** Jill Barr-Walker, Iesha Nevels

**Affiliations:** ZSFG Library, University of California, San Francisco, San Francisco, CA; ZSFG Library, University of California, San Francisco, San Francisco, CA

## Abstract

**Background:**

Hospital libraries must often demonstrate value to users who are not aware of their services. Zuckerberg San Francisco General Hospital (ZSFG) Library aimed to increase patient and staff awareness using innovative outreach methods through our involvement in a Summerfest health fair and a National Medical Librarians Month event.

**Case Presentation:**

At 2 hospital events, ZSFG Library staff and volunteers used a game show–style approach involving active learning to teach attendees about library resources and services. Across events, there were 300 attendees and 167 in-depth discussions of library resources with the librarian, including 54 demonstrations. After implementing these new outreach efforts, the number of attendees increased by over 240% and meaningful interactions increased by 1,300% from the previous year’s event. Our value analysis indicates an overall positive effect with 14 minutes of total library staff time spent per meaningful interaction.

**Conclusions:**

The use of a spinnable wheel for asking participants library-related questions and a television monitor to demonstrate library resources greatly increased the number of attendees and fostered new staff connections, resulting in several in-service trainings and search requests. Future recommendations for outreach events include enlisting the help of volunteers to record attendance data, creating materials in multiple languages, and integrating library involvement into existing hospital events. These recommendations may decrease the amount of library staff time spent in return for each meaningful interaction, creating increased value for less time.

## BACKGROUND

Hospital libraries are constantly seeking ways to demonstrate value in order to avoid the dangers of closures and budget cuts, and this often requires venturing outside the library and forming relationships with staff in other parts of the hospital. As a result, health sciences librarians often engage in outreach work that targets clinical staff, patients, and members of the community to raise awareness of library services. Some innovative outreach efforts have included creating web portals, utilizing social bookmarking tools and online guides, hosting story times for children in waiting rooms, and creating a traveling library to promote services to off-site staff [1–5]. Some medical libraries have successfully created targeted outreach efforts for specific populations including support groups, outpatient visitors, and older adults [4–6].

Health fairs are outreach events that public, consumer health, and hospital libraries often use to provide opportunities to promote health literacy among patients and community members, learn more about the needs of patients and staff, form community partnerships, and increase the visibility of the library [7–9], although some have noted concerns about the value received for the time spent on these events [10]. This case study investigates the library’s involvement in two hospital outreach events by presenting a value analysis for staff time spent and meaningful interactions received.

Zuckerberg San Francisco General Hospital and Trauma Center (ZSFG) is an urban safety net hospital in San Francisco that provides inpatient, outpatient, emergency, diagnostic, and psychiatric services for adults and children. ZSFG’s strengths include a commitment to serving vulnerable populations, internationally known HIV/AIDS research and care, and status as the only Level I trauma center in San Francisco. The hospital employs 5,300 people, about one third of whom are University of California, San Francisco (UCSF) employees, including all physicians, residents, and researchers. Most other clinical staff are employed by the San Francisco Department of Public Health. ZSFG Library serves all hospital staff, and because of the multi-institutional nature of the hospital and the fact that no all-staff directory or email list exists, it is often difficult to plan targeted outreach to our user population.

The clinical librarian at ZSFG is responsible for all reference, outreach, and research assistance for hospital staff. In the past, the librarian was able to build relationships with UCSF researchers and clinicians, as these groups were interested in workshops focused on literature searching and data management, but she struggled to reach Department of Public Health staff and patients.

Summerfest at ZSFG is a health fair for ZSFG staff members, patients, and the public aiming to celebrate ZSFG’s diversity while creating a culture of wellness. In 2016, the clinical librarian (Barr-Walker) and library assistant (Nevels) were invited to participate in this event with a table that contained basic handouts. We received fewer than fifty visitors at our table, and only three of these visitors engaged in what were considered meaningful interactions, defined here as an in-depth conversation in which the librarian and patron discussed library services, including requests for information that led to follow-up reference requests. Because of the small turnout and lack of interactions at our table, we decided to create an interactive display the following year, based in part on a raffle display that we observed during ZSFG Patient Safety Week. We decided to try our new idea at two outreach events the following year.

## STUDY PURPOSE

ZSFG Library staff were involved in two outreach events in 2017: ZSFG Summerfest in June 2017 and the library’s own event for National Medical Librarians Month in October 2017. Our primary aim was to use these two events to increase patient and staff awareness of ZSFG Library in a way that encouraged communication, engagement, and active learning. Our objectives for these events were as follows: (1) connect patients, hospital staff, and community members to health information services and resources and inspire wellness behaviors to promote a healthy lifestyle; (2) increase awareness of ZSFG Library resources (e.g., MedlinePlus, PubMed, and DynaMed) around major health topics, with particular focus given to vulnerable, ethnically diverse, and underserved populations of San Francisco; and (3) increase patient and staff awareness of ZSFG Library, the library staff, and library resources in an entertaining way.

## CASE PRESENTATION

### Preparation for the events

We received a National Network of the Libraries of Medicine (NNLM) mini-outreach grant for $1,206 to assist with purchasing supplies for the outreach events. Using this grant, we purchased a 50-inch television monitor and stand, an upright spinnable wheel, and several $10 gift certificates for a convenience store near the hospital. The clinical librarian and library assistant were responsible for planning the library’s participation in both events.

### The events: Summerfest and National Medical Librarians Month

**Figure 1:**
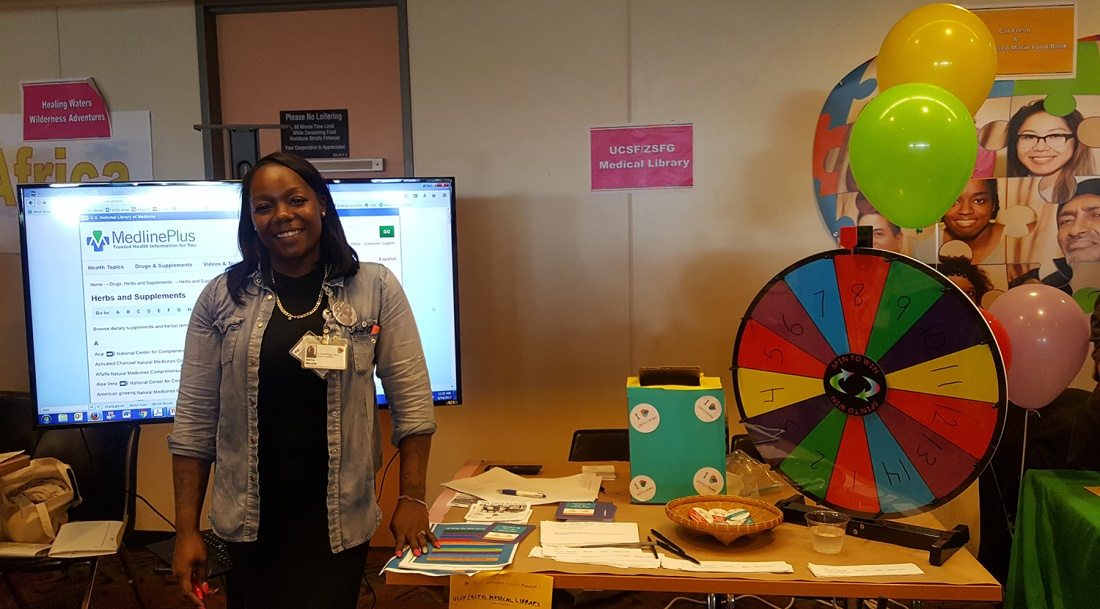
Zuckerberg San Francisco General Hospital Library assistant, Iesha Nevels, and the setup for Summerfest

The clinical librarian and library assistant staffed a table together at each event. We set up a colorful upright spinning wheel, paper slips and a container for the raffle drawing, and a laptop computer and large monitor to demonstrate databases and National Library of Medicine (NLM) resources (Figure 1). When someone approached our table, we asked if they would like to enter our raffle. To enter, they would need to answer a question about ZSFG Library. Participants spun the wheel, which landed on a number; each number corresponded to a question on a list, and that question was asked. There were two lists of questions: one for ZSFG staff and one for patients and members of the public. A complete list of questions is shown in Table 1.

**Table 1:** List of questions used in the raffle activity for Summerfest and National Medical Librarians Month events

Questions for staff		Questions for patients and the public	
**Summerfest**			
1.	Where is the library? How can you reach us if you have a question?	1.	Where is the library? How do you reach us if you have a question?
2.	Is the library open to the public?	2.	Is the library open to the public?
3.	What are the library hours?	3.	What are the library hours?
4.	Name one point of care resource you can use via the library.	4.	True or false: I can check my email in the library.
5.	Name a place to find examples of clinical best practices and guidelines.	5.	True or false: If I want to find out more information about my treatment or diagnosis, I can ask the library.
6.	How can the library help with QI projects and Lean projects?	6.	Where can you find information about preventing diabetes?
7.	Name an example of something you can find in the library archives.	7.	Where can you find information about healthy recipes?
8.	True or false: If you can’t find a PDF online, the library will find it for you.	8.	Where can you find information about complementary and alternative medicine?
9.	Name one way the library can help your patients.	9.	Where can you find health information in Spanish, Chinese, Russian, Tagalog, and other languages?
10.	True or false: The library is here to save me time!	10.	Where can you find information about clinical trials in the Bay Area?
**National Medical Librarians Month event**			
1.	Where is the library? How can you reach us if you have a question?	1.	Is the library open to the public?
2.	Name one point of care resource you can use via the library.	2.	What are the library hours?
3.	Name one way the library can help your patients.	3.	Where can you find information about preventing diabetes and healthy recipes?
4.	Name an example of something you can find in the library archives.	4.	True or false: If I want to find out more information about my treatment or diagnosis, I can ask the library.
5.	True or false: If you can’t find a PDF online, the library will find it for you.	5.	True or false: I can check out books from the library.

Each question was designed to promote library services, collections, and spaces in an engaging way. Our goal was not to test participants’ knowledge; rather, we wanted to share information about the library using active learning techniques in a nonjudgmental way and to show participants that ZSFG Library staff were approachable and knowledgeable. After participating in this activity, participants could share their contact information to be entered into our raffle for a gift card.

Both the librarian and library assistant assisted attendees with spinning the wheel, asked questions, and provided answers to the questions. When appropriate, we demonstrated library resources using the large monitor. The most commonly demonstrated resources were DynaMed, a point-of-care tool used by clinicians, and MedlinePlus, a consumer health resource for patients. We highlighted these particular tools because of the hospital’s recent transition from UpToDate to DynaMed, the lack of knowledge about these tools among ZSFG staff, and the non-English language materials in these tools that reflect the needs of our patient population.

At the Summerfest event, in addition to the spinning wheel raffle, we aimed to administer surveys to every tenth attendee, which asked about their current library use and information needs. At the National Medical Librarians Month event, in addition to the activities mentioned above, we organized a mini pop-up event with the UCSF Makers Lab to demonstrate button making, 3D printing, and other services.

## RESULTS

### Summerfest

Summerfest was a 3-hour event held on a Friday from 11:00 a.m. to 2:00 p.m. Although the total number of attendees was not counted, our raffle received 120 entries, including 68 staff members, 32 patients, and 20 members of the public. The librarian had 42 meaningful interactions, including 15 demonstrations using the television monitor. The librarian received 4 requests for presentations at staff meetings, 3 requests for in-service trainings, and 2 requests for searches. Each of these requests was received from groups that the librarian had not previously worked with, including a primary health outpatient clinic, the outpatient pharmacy department, and the nutrition department. In comparison to our 2016 Summerfest event, our event using new outreach methods received a 240% increase in participants and a 1,300% increase in meaningful interactions.

### National Medical Librarians Month

The National Medical Librarians Month event was held on a Monday, Wednesday, and Friday at lunchtime (11:00 a.m.–1:00 p.m.). Across the 3 days, there were 181 attendees and 119 raffle entries, including 99 staff members, 8 patients, and 12 members of the public. The librarian had 125 meaningful interactions, including 39 demonstrations. There were 5 requests for presentations at staff meetings, 1 in-service training request, and 1 search request, all from unique and new-to-the-library groups, such as interpreter services, social services, and a reproductive health clinic for teens.

### Measuring value

We used our results data to conduct an informal value analysis that measured the amount of library staff time spent on preparing and conducting the events and the number of meaningful interactions with patrons. Two library staff members prepared for the events, including applying for the NNLM grant; selecting, purchasing, and assembling materials; creating event content including questions, raffle slips, and surveys; creating and disseminating promotional materials; evaluating post-event data; and liaising with multiple departments including Facilities, Food Services, Hospital Administration, and the Makers Lab. We estimated this preparation time to be 18.5 hours. In addition, 2 library staff members each spent 10.5 hours at the events for a total of 39.5 hours for 2 events. By dividing these hours by 167 meaningful interactions, we estimated that approximately 14 minutes of library staff time were spent per meaningful interaction.

## DISCUSSION

### Successes

We considered our outreach events to be successful as measured by the number of people we reached and follow-up requests that the librarian received. The spinning wheel was instrumental in our success; not only was it an eye-catching way to draw people to our table, it was also a fun, low-pressure way to teach people about the library’s services.

Our raffle questions were an engaging way to promote library services while employing active learning techniques to teach participants about information resources. Between the first and second events, we adjusted the questions slightly, to five options each for staff and patients so that they would all be easily readable on one sheet of paper for each of us to carry. These five questions focused on the main information that we wanted to convey: point-of-care resources, patient information, library archives, and our location and hours. We found that having the librarian focus on staff interactions and the library assistant focus on patient and public interactions worked well for us, as these divisions reflected our job roles and interests.

The number of visits received and meaningful interactions experienced suggest that our approach was successful in increasing awareness of ZSFG Library among hospital staff, patients, and members of the public. As a result of connections made at these events, the librarian has presented at several staff meetings, conducted many in-service trainings, and received numerous questions from departments that were previously underserved by the library. The resources showcased during the events, especially the DynaMed mobile app and the non-English-language materials on MedlinePlus, were appreciated by staff, most of whom expressed their intention to use these resources in the future.

One of the biggest successes from the librarian’s perspective was the help of the library assistant. Without her efforts designing the raffle materials, liaising with cafeteria staff to plan the events, and engaging with patients and members of the public at our tables, these events would not have been possible. Enlisting the help of dedicated staff is crucial to the success of outreach events and makes the events more enjoyable for everyone involved. Although we did not collect data for the library assistant’s meaningful interactions, we will consider doing so in the future to capture a more complete representation of the contributions of all library staff at outreach events.

As others have noted, health fairs can provide an opportunity to improve health literacy and awareness of health information resources [9]. An unexpected success of our event was an increased knowledge of library resources by library staff. In preparation for these events, the librarian provided demonstrations of DynaMed and MedlinePlus to library staff so they would be able to answer questions about these resources. Library staff have since reported feelings of empowerment around using these resources to help library patrons and answer their own questions about health topics. As a result, the librarian now offers regular training sessions about library resources for library assistants.

### Lessons learned

#### Staffing the table

For our NNLM grant, we were required to record data about the number of participants, demonstrations, and meaningful interactions. At the Summerfest event, with only 2 people staffing our table and over 100 attendees in 3 hours, we were unable to record these data in a consistent way; therefore, we judged the overall attendance by the number of people who entered the raffle. At the National Medical Librarians Month event, we enlisted the help of hospital volunteers to keep track of these data. In addition to providing this helpful service, they acted as unexpected cheerleaders, encouraging staff to stop by our table and sharing information about library services. Some volunteers learned about ZSFG Library for the first time during the event. We recommend using volunteers or clickers to record attendance data as it is not possible to do this consistently when staffing a busy table.

#### Materials

We were surprised by the lack of knowledge about MedlinePlus among staff and patients and promoted it using live demonstrations whenever possible. At our second event, we passed out bookmarks containing the MedlinePlus website information for attendees to reference later. Collecting data on attendees’ knowledge of and comfort with MedlinePlus or other library resources, as has been done in other outreach settings [6], may help library staff better understand our users’ consumer health information needs.

Another unexpected finding was the large number of non-English speaking attendees, who represented the demographics of our patient population. We were able to communicate in basic Spanish with some attendees and demonstrated MedlinePlus en Español, but many Summerfest attendees were unable to fill out raffle slips to receive more information. In the future, we will work with the hospital’s translation services to develop materials and raffle slips in other languages, primarily Spanish and Chinese.

#### Survey

We were interested in learning about library needs from our staff, patients, and members of the public. However, asking attendees to fill out a survey in the short time that they were visiting our table was not feasible. Based on participants’ answers to the raffle questions, it was apparent that many did not know the library existed; this information itself is valuable for our outreach efforts. The librarian was able to have longer conversations with some staff members about their library needs, which replaced the need for surveys in many cases.

#### The raffle

We found that people enjoyed participating in a raffle without placing much value on the nature of the prize. At Summerfest, because we had not yet purchased the prize, we were purposely vague in saying that winners would win a gift card (later revealed as a convenience store gift card). This did not deter anyone from participating. In fact, we had a difficult time getting winners to claim their prizes after the event. Contacting ZSFG staff members proved especially difficult, as we learned that many clinicians do not check their email regularly. We eventually hand-delivered the prizes based on departmental information collected on the raffle slips, which proved to be an additional way to meet staff who do not visit the library regularly. At the second event, in addition to the raffle drawing held after the event, we handed out gift cards to one attendee every thirty minutes, and we found this method of distributing prizes to be a better fit for our hospital.

#### Event planning

We learned that integrating into an existing hospital event is preferable to organizing a separate library event and will continue to work with ZSFG’s employee recognition committee to integrate our table into the hospital’s holiday party, fall festival, and other hospital-wide events. We also learned that a one-day event is more manageable than multiple-day events. We learned that, given a choice of day, Friday at lunchtime will bring attendees who are more receptive to spending a few minutes to learn about library services: Friday’s attendance at our event was five times that of Monday.

### Measuring value

Some libraries have noted that the inability to devote sufficient time to planning and participating in health fairs may be prohibitive to being involved in these events [10]. The majority of our library staff time was spent in preparation for the events: selecting and purchasing materials, applying for the grant, and designing event and promotional materials. Because most of these are one-time costs, we believe that this type of outreach event is sustainable, and we can use our existing materials and equipment for future events with minimal preparation time needed. Integrating library outreach into existing events will also reduce preparation time as library staff will not need to communicate with multiple departments to coordinate space or disseminate promotional materials. Implementing the above recommendations will likely decrease library staff time spent in return for each meaningful interaction, ultimately creating greater value for less time spent.

## CONCLUSION

The outreach events met our overall objective of increasing patient and staff awareness of ZSFG Library’s resources and services in an entertaining way. With the use of eye-catching materials like a spinning wheel and television monitor, we increased visibility of the library and had fun doing it. We hope to continue to use these low-cost, high-value, sustainable methods at health fairs and outreach events in the future to promote ZSFG Library services and resources to our patient and staff community.
